# Treatment of a Vertical Root Fracture Using Dual-Curing Resin Cement: A Case Report

**DOI:** 10.1155/2012/985215

**Published:** 2012-12-18

**Authors:** Nima Moradi Majd, Farshid Akhtari, Solmaz Araghi, Hamed Homayouni

**Affiliations:** ^1^Department of Endodontics, Dental School, Qazvin University of Medical Sciences, Qazvin 34157-59811, Iran; ^2^Iranian Center for Endodontic Research, Dental Research Center, Dental School, Shahid Beheshti University of Medical Sciences, Tehran 19839-63113, Iran; ^3^Dental Research Center, Shahid Beheshti University of Medical Sciences, Tehran 19839-63113, Iran

## Abstract

*Introduction*. Vertical root fracture (VRF) is one of the most frustrating complications of root canal treatment. The prognosis of the root with VRF is poor therefore tooth extraction and root amputation are usually the only treatment options. However, bonding of the fracture line with adhesive resin cement during the intentional replantation procedure was recently suggested as an alternative to tooth extraction. *Methods*. A vertically fractured left maxillary incisor was carefully extracted, fracture line was treated with adhesive resin cement, a retrograde cavity was produced and filled with calcium-enriched mixture (CEM) cement, and tooth was replanted. *Results*. After 12 months the tooth was asymptomatic. The size of periapical radiolucency was noticeably reduced and there was no clinical sign of ankylosis. *Conclusion*. Using adhesive resin cement to bond the fracture lines extraorally in roots with VRF and intentional replantation of the reconstructed teeth could be considered as an alternative to tooth extraction, especially for anterior teeth.

## 1. Introduction

An obliquely or longitudinally oriented breakage of the root that originates from the apex and propagates coronally is called vertical root fracture (VRF) [1]. Indeed, VRF is one of the most infuriating undesirable consequences of root canal therapy, because it results in the tooth or root extraction [2]. It was reported that the prevalence of VRF lies in the range of 11%–20% in extracted endodontically treated teeth [3, 4].

As described previously [5] the most important risk factors for VRFs include overpreparation of root canal and post space, excessive lateral and vertical compaction forces during obturation, moisture loss in endodontically treated teeth, and superfluous pressure during post placement.

Although several solutions have been suggested for managing VRF cases, such as tooth extraction and implant replacement [6], root resection using a surgical method [7], and utilizing CO_2_ laser to fuse VRF [8], no specific treatment modality has been established [9]. VRF reconstruction with adhesive resin cement has shown successful outcome in several studies [9–13]. According to long-term clinical results, bonding of fractured segments after tooth extraction with an adhesive resin cement and then replantation of the tooth could be an alternative approach to extraction [12, 13].

The present case report describes a successful treatment of a VRF using adhesive resin cement to fill the outer space of the fracture line.

## 2. Case Presentation

**Figure 1:**
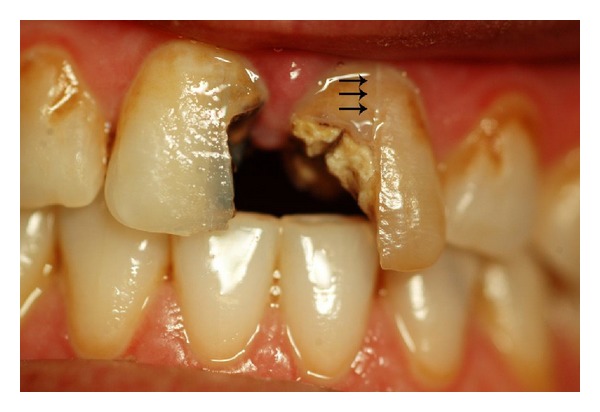
Preoperative photograph; arrows showing the crack line on the facial surface of left maxillary central incisor.

**Figure 2:**
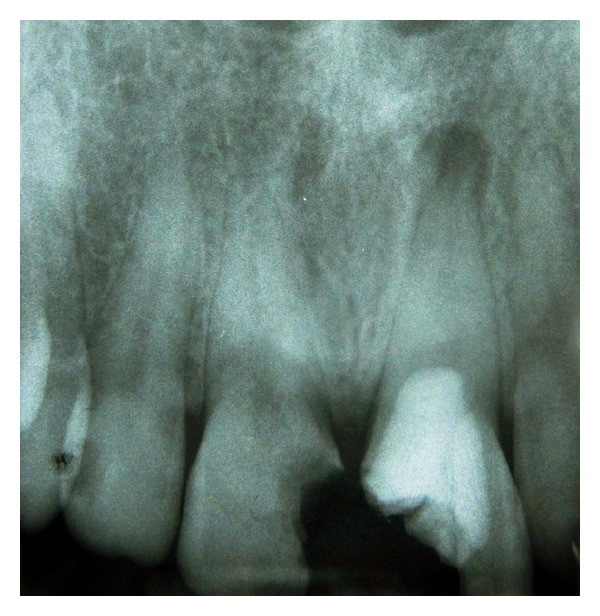
Preoperative radiograph; root canal of the left maxillary central incisor has been over-prepared and periapical radiolucency is observed at the apex of the tooth.

A 36-year-old female with no contributing medical history was referred to the Endodontic Department of Qazvin School of Dentistry. She proclaimed that her left maxillary central incisor underwent incomplete root canal therapy five months ago. She stated that she could not continue the treatment due to financial problems. After clinical examination, a longitudinal fracture on the facial surface of the tooth was observed; also a solitary pocketwith a depth of 8 mm was detected in the facial region (Figure 1). Radiographic examination revealed that the canal has been over-prepared during previous endodontic treatment but it has not been obturated. In addition, periapical radiolucency was observed at the apex of the left maxillary incisor (Figure 2). The presence of a deep pocket along a longitudinal fracture convinced us to extract the tooth and treat the patient with an implant placement, but as was mentioned above the financial problems were the main patient's limitation for the desired treatment plan. 

In view of this limitation we decided to extract left maxillary incisor, reconstruct the fracture with adhesive resin cement, perform a root end resection, prepare a retrograde cavity and fill it using filling material, and replant the tooth. A written informed consent was obtained and the patient was scheduled for treatment.

**Figure 3:**
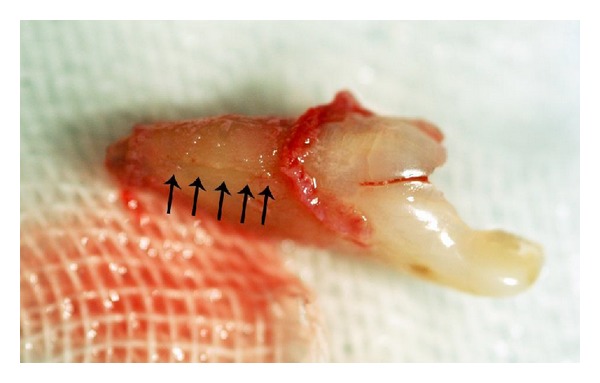
Arrows showing the fracture line on the buccal surface of the root.

At the patient's return, antisepsis was carried out using 0.2% chlorhexidine gluconate; left maxillary incisor was anesthetized using an infiltration and nasopalatine nerve block injection (lidocaine 2% with epinephrine 1 : 80000; Daroupakhsh, Tehran, Iran). The tooth extraction was gently performed using forceps with no intraoperative complications; subsequently tooth structure was carefully examined and it was found that fracture line has been originated from the apex and propagated coronally (Figure 3). Extracted tooth was kept in moistened gauze using normal saline, and in order to remove inflamed tissue, socket walls adjacent to the fracture region were curetted and irrigated again using normal saline. 

**Figure 4:**
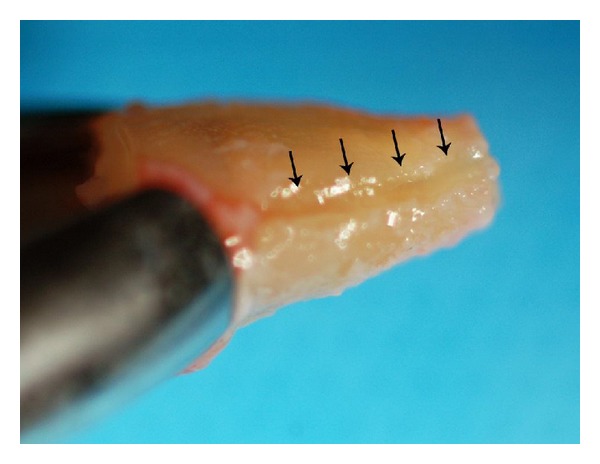
Arrows showing the shallow preparation of the fracture line.

**Figure 5:**
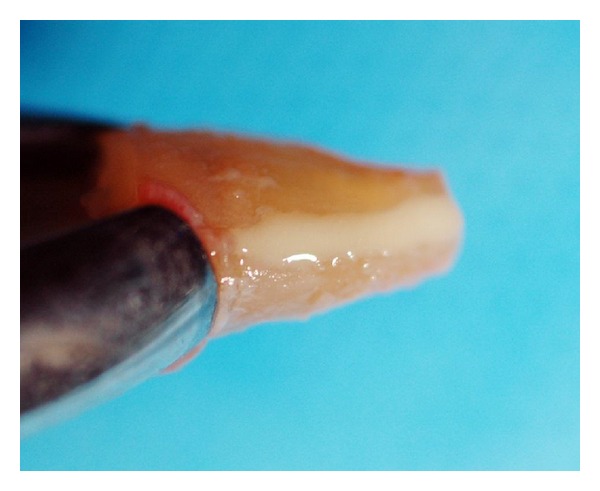
The prepared fracture line was sealed with dual-curing resincement.

**Figure 6:**
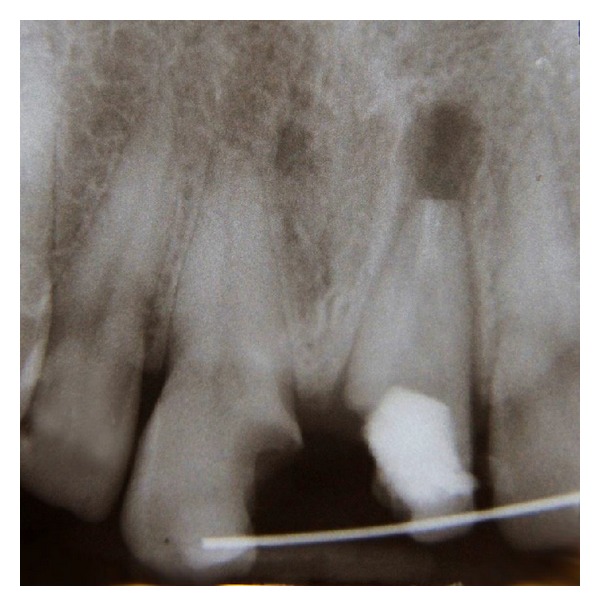
The tooth was immobilized after replantation.

**Figure 7:**
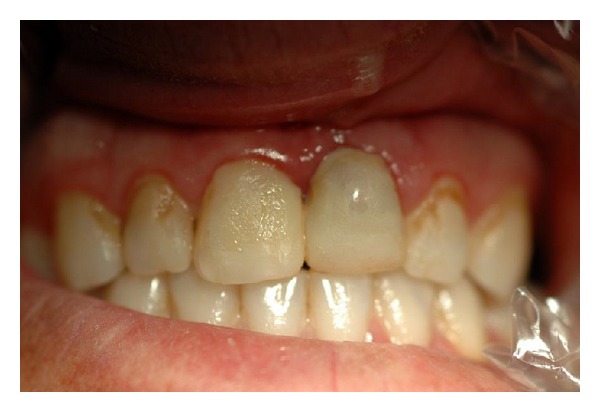
Photograph at fifth week after replantation; right and left maxillary incisors were restored.

A shallow preparation of the fracture line, root end resection, and removal of the resorptive defect of root's apical portion were extraorally carried out (Figure 4). Afterward, the prepared fracture line was sealed with dual-curing resincement (Panavia F, Kuraray Co. Osaka, Japan); minimal cement was applied to avoid covering the periodontal ligament (Figure 5); the resin was cured for 20 seconds with a light-curing unit (Degulux; Degussa AG, Frankfurt, Germany). The root end cavity was prepared and filled with calcium enriched mixture (CEM) cement (BioniqueDent, Iran). In order to enhance periodontal ligament cell attachment, the root surfaces were treated with tetracycline for 30 seconds [14]. The extracted tooth was then replanted in its original position [9–13]. The whole procedure lasted 18 minutes. After replantation, the tooth was immobilized with a semirigid splint for 10 days (Figure 6). Chlorhexidine gluconate mouth rinse and 4 × 400 mg ibuprofen plus 3 × 500 mg amoxicillin daily for a week were prescribed. Five weeks after replantation, the tooth was temporally restored with a fiber post-retained composite restoration (Figure 7).

## 3. Clinicoradiographical Followup

**Figure 8:**
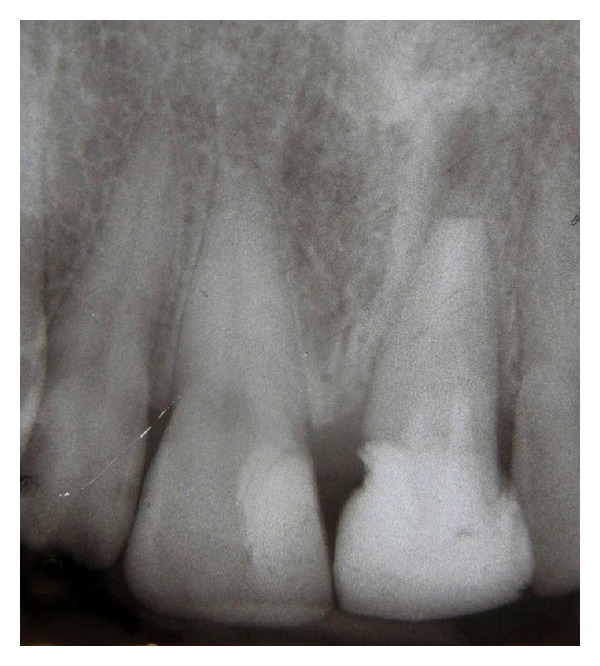
12 months after intentional replantation; periapical radiolucency was noticeably reduced.

Tooth mobility and sensitivity to percussion were examined every three months. The percussion tone was evaluated and compared with adjacent teeth. At 12 months after intentional replantation, the tooth was completely asymptomatic, with physiologic mobility. Furthermore, periapical radiolucency was noticeably reduced (Figure 8). 

After one year followup, in order to restore the tooth with full-cast crown, the patient was referred to prosthetic department. 

## 4. Discussion

The prognosis of the root with VRF is poor therefore tooth extraction and root amputation are usually the only treatment options [2], but recently, several attempts have been made to save the fractured roots from extraction [8–13]. One of the innovative methods that provide an alternative to tooth extraction, especially for anterior teeth is extraoral bonding of the extracted fragments with adhesive resin cement and intentional replantation of the reconstructed tooth [9]. Although, Hayashi et al. [10] reported that no failure was observed in vertically fractured incisors treated with this method, they stated failures did occur in premolars and molars which are treated using reconstruction with adhesive resin cement. Özer et al. [9] suggested two main reasons for positive outcomes of this method in anterior teeth: (1) the posterior teeth were negatively affected by strong occlusal forces. (2) The morphology and location of anterior teeth facilitate the maintenance of gingival health. In addition, Arıkan et al. [11] reported that this method had a successful outcome for VRF treatment, and they recommended the procedure. They also stated that in order to preserve the vitality of the periodontal ligament, and increase the probability of long-term replantation success, extraoral working time should be shortened. They then showed that the use of a dual-curing material reduced the amount of time that was extraorally spent. On the basis of these studies and the good prognosis they reported, the present case was referred for intentional replantation after reconstruction with dual-curing resincement.

As was described previously [5] one of the most important risk factor for VRF is excessive lateral and vertical compaction forces during obturation. Therefore, in order to avoid imposing wedging forces, tooth was retrofilled instead of conventional obturation with gutta-percha and sealer. In so doing, the root of the central incisor was resected and retrofilled using CEM cement. CEM cement is a biocompatible biomaterial [15] which is showed that has a good sealing ability when it used as a root-end filling material [16]. It is also demonstrated that CEM cement's apical plug has superior sealing ability compared to MTA plug [17]. On the basis of these findings, in this case CEM cement was used as a retrograde filling material.

The most important factors in prevention of ankylosis are the presence of healthy cementum on the root surface and periodontal ligament vitality [18]. In order to modify root surface and produce a surface that is conductive to cellular adhesion and growth, several solutions have been advocated, for instance using tetracycline, citric acid, and ethylene diamine tetra-acetic acid (EDTA) [19]. Madison and Hokett [14] reported that a 30-second application of tetracycline can successfully remove the smear layer. Also, Özer et al. [9] applied tetracycline to their subjected teeth for thirty seconds just before the teeth replantation. Thus, in order to enhance periodontal ligament fiber attachment, and prevent ankylosis, tetracycline was applied to the root surfaces, in this case too.

According to previous study [20], ankylotic areas of teeth can be observed radiographically when the ankylosis was located on the proximal surfaces of the root, but if they occur on the lingual or facial surfaces, they will not be detected. Therefore, considering the fact that the initial location of ankylosis is often on the labial and/or lingual root surfaces [20, 21], radiographic examination cannot be a reliable approach to early detection of ankylosis because theradiographic image corresponds to a two-dimensional aspect of a three-dimensional structure [21].

Thus, the evaluation of mobility and percussion sound as we performed during our controls could be more helpful to detect ankylosis. 

After 12 months the subjected tooth was mobile within normal limits, and the percussion tone was the same as that of the healthy adjacent tooth. Although monitoring a replanted tooth for a long period of time is favorable, the absence of ankylosis for a mean period of one year suggests a good long-term prognosis [9].

## 5. Conclusion

Using adhesive resin cement to bond the fracture lines extra-orally in roots with VRF and intentional replantation of the reconstructed teeth could be considered as an alternative to tooth extraction, especially for anterior teeth.
